# News and Events of the Week

**Published:** 1909-10-09

**Authors:** 


### News and Events of the Week.

A "somewhat serious" epidemic of scarlet fever is reported from Glasgow. No less than 744 cases have been notified in one week.

The West Bristol Liberal Executive has recommended Dr. Walter Saise as Liberal candidate in opposition to the sitting Unionist member, Mr. G. A. Gibbs.

The number of deaths at Holborn from phthisis during the past year was 105, or a death rate of 1.89 per 1,000, the lowest recorded for the borough. The corrected number of deaths in London was 6,419, equal to a rate of 1.32 per 1,000, being the lowest recorded.

Professor Theodor Schott, of Nauheim, will deliver an address at the Postgraduate College, West London Hospital, Hammersmith Road, W., on Monday, October 11, at 5 p.m. The address will deal with "Renewed Research on the subject of Acute Overstraining of the Heart."

At the last meeting of the Penzance Board of Guardians the medical officer reported that thirteen of the children in the workhouse required dental attention, and recommended that tooth-brushes be supplied to all the children in the House. The report was referred back to the doctor.

An "organised games playground" was recently opened at Goldsmith Square, Shoreditch. The average daily attendance of children during the school holidays has been over a thousand. The Borough Council is now urging the adaptation of the picturesque Ironmongers' Almshouses in Kingsland Road as an open-air school.

The Birmingham Health Committee, anxious to secure for the city a milk supply free from tuberculosis, has sanctioned a scheme for giving free of charge the necessary tuberculin and veterinary assistance for the testing of cows twice annually. Circulars giving details of the scheme are being issued to the farmers concerned.

Dr. G. Wilkinson has recently described how it is sometimes possible in non-Christian countries to gain the loan of a temple for hospital purposes. An instance of this occurred at Dusung, in China, where for a fortnight opium patients were allowed to be treated medically through the mission there.

The prize of 1,000 crowns offered by the Hungarian Minister of the Interior for the best work on Trachoma, which was to have been given at the International Congress of Medicine at Budapest, has been withheld. In the opinion of the jury, none of the essays sent in, seven in number, indicated any remarkable progress.

When Dr. J. Jamieson resigned his position as lecturer in medicine at the Melbourne University last year, after continuous service of 30 years, past and present students decided to make him a presentation. About £150 has been subscribed, and at Dr. Jamieson's request the money is to be devoted to founding an annual prize at the final examination in clinical medicine at the University.

Mr. Charles Carty Salmon, the new Federal Speaker, is a native-born Australian who, adopting medicine as a profession, took the qualifications of L.R.C.P. *et* S., and L.F.P.S. at Edinburgh. Entering politics in 1893, he was in that year elected member of the Victorian Legislative Assembly for Talbot and Avoca, which seat he continued to represent until 1901, when he became a member of the Commonwealth House of Representatives.

Owing to the unrest in Persia, the departure of several medical missionaries, who would under normal circumstances have returned in the spring, was postponed. The situation has now so far improved that two parties proceeding to Persia, include the following medical workers: Dr. and Mrs. H. White, Dr. Emmeline M. Stuart, Dr. Winifred A. Westlake, Dr. Alicia P. Aldous, Miss A. Macklin, and Miss H. McKim left for Persia last month.

Dr. G. S. Seccombe has retired from the post of Lunatic Asylum Superintendent, St. Ann's, Trinidad.

Dr. J. Tichborne, Medical Officer of Southern Nigeria, has been transferred to the Gold Coast Colony in a similar capacity.

The prosperity of Australia continues to attract increasing numbers of settlers to her shores. The new Orient liner *Otranto,* whose magnificent decoration and proportions have been already noticed in these columns, created a new record last week with a list of more than 1,100 passengers in all classes.

A decrease has taken place in the mortality of the country. The Registrar-General's returns state that during the week ended September 25 the average death-rate in the 67 principal provincial towns of England and Wales was 12.6 per 1,000, as against 13.0 in the previous week.

For ten years the Surbiton District Council has been discussing how to dispose of its sewage. At present the sewage of part of the district is treated by the Kingston Corporation, who have offered to take the whole of the sewage on favourable terms, and this course seems likely now to be adopted.

Lady Dimsdale has agreed to accept the position of Lady President of a City of London branch of the League of Mercy, which is about to be constituted, with the Lord Mayor for the time being as president. A meeting in support of the movement is to be held at the Mansion House on Thursday, October 21.

The third international Congress on School Hygiene will meet in Paris from March 29 to April 2, 1910. The Congress will be under the presidency of the French Minister of Public Instruction. Conferences are triennial, the previous having been held at Nuremberg in 1904 and at London in 1907. An international exhibition of school hygiene will be held in Paris at the same time as the Congress.

An important conference will be held in Han-kow under the auspices of the China Medical Missionary Association at the Chinese New Year, 1910. The programme is being arranged by a committee of eight doctors, one of whom is Dr. A. F. Cole, of the C.M.S. Ningpo Medical Mission, who is in charge of the surgical section. Medical education in China is now a problem pressing for solution, which the conference should help to further in many ways.

The John Morley Chemical Laboratories, an important addition to the buildings of Manchester University, were opened on October 4th by Sir Henry Roscoe, for many years the Professor of Chemistry of the University. The new premises include a spacious laboratory for organic chemistry, research rooms, private laboratory, and accommodation for the professors. For the third-year students there is a large laboratory on the ground floor.

The Incorporated Institute of Hygiene, 34 Devonshire Street, Harley Street, W., has just issued a syllabus of a course of 32 popular lectures, with practical demonstrations on Food and Dietetics, Cooking, Home Hygiene, and Home Nursing, to be given in the lecture hall of the Institute at 3.30 p.m. during the next eight months. One lecture on each of the above four groups of subjects will be given each month in the order named. The first lecture on Cooking was delivered on Tuesday, October 5. The complete syllabus will be sent on application to the Secretary.

